# Downregulation of CD9 in Keratinocyte Contributes to Cell Migration via Upregulation of Matrix Metalloproteinase-9 

**DOI:** 10.1371/journal.pone.0077806

**Published:** 2013-10-16

**Authors:** Xu-pin Jiang, Dong-xia Zhang, Miao Teng, Qiong Zhang, Jia-ping Zhang, Yue-sheng Huang

**Affiliations:** 1 Institute of Burn Research, State Key Laboratory of Trauma, Burns and Combined Injury, Southwest Hospital, The Third Military Medical University, Chongqing, China; 2 Department of Burn and Plastic Surgery, The First Affiliated Hospital of Chongqing Medical University, Chongqing, China; UMR CNRS 5242 - ENS de Lyon- Université Lyon 1, France

## Abstract

Tetraspanin CD9 has been implicated in various cellular and physiological processes, including cell migration. In our previous study, we found that wound repair is delayed in CD9-null mice, suggesting that CD9 is critical for cutaneous wound healing. However, many cell types, including immune cells, endothelial cells, keratinocytes and fibroblasts undergo marked changes in gene expression and phenotype, leading to cell proliferation, migration and differentiation during wound repair, whether CD9 regulates kerationcytes migration directly remains unclear. In this study, we showed that the expression of CD9 was downregulated in migrating keratinocytes during wound repair *in vivo* and *in vitro*. Recombinant adenovirus vector for CD9 silencing or overexpressing was constructed and used to infect HaCaT cells. Using cell scratch wound assay and cell migration assay, we have also demonstrated that downregulation of CD9 promoted keratinocyte migration *in vitro*, whereas CD9 overexpression inhibited cell migration. Moreover, CD9 inversely regulated the activity and expression of MMP-9 in keratinocytes, which was involved in CD9-regulated keratinocyte migration. Importantly, CD9 silencing-activated JNK signaling was accompanied by the upregulation of MMP-9 activity and expression. Coincidentally, we found that SP600125, a JNK pathway inhibitor, decreased the activity and expression of MMP-9 of CD9-silenced HaCaT cells. Thus, our results suggest that CD9 is downregulated in migrating keratinocytes *in vivo* and *in vitro*, and a low level of CD9 promotes keratinocyte migration *in vitro*, in which the regulation of MMP-9 through the JNK pathway plays an important role.

## Introduction

CD9 is a member of the tetraspanin superfamily of integral trans-membrane proteins comprising of four transmembrane domains, two extracellular loops and short intracellular ends [1,2]. Although originally identified in the cells derived from the hematopoietic lineage, CD9 is also constitutively expressed in a diverse variety of other cell types [2,3] to facilitate various cellular and physiological processes, including cell motility [4], migration and adhesion [5,6]. The inhibition of CD9 using anti-CD9 monoclonal antibody or siRNA led to enhanced motility of the primary human melanocytes [7]. Additionally, CD9 could negatively regulate the migration of Schwann cells and endothelial cells [8,9]. However, the reduction of CD9 caused an inhibition of melanoma cell motility [10] whereas the transient overexpression of CD9 in human metastatic melanoma cells resulted in an increase of matrigel invasion activity [11]. These previous observations indicate that the role of CD9 in cell migration and motility is not consistent among different cell types.

Wound healing represents a dynamic and well-ordered biological process [12]. Re-epithelialization, an essential step for wound healing, involves the migration of epidermal keratinocytes over the wound site. CD9 has been reported to be expressed in skin epidermis [13]. Our previous *in vivo* study revealed that CD9 is critical for cutaneous wound healing as wound repair is delayed in CD9-null mice [14]. However, various intracellular and intercellular pathways must be activated and coordinated after an injury. Besides, many cell types, including immune cells, endothelial cells, keratinocytes and fibroblasts undergo marked changes in gene expression and phenotype, leading to cell proliferation, migration and differentiation [15,16]. While healing delay resulting from impaired migration of the epidermis was observed in the model of CD9 knockout mice used for our previous study [14], we were unable to exclude the possibility of functional compensation that may occur in a knockout mouse which may mask or distort the phenotype resulting from the chronic absence of an endogenous gene. Therefore, it remains unclear whether CD9 plays a role in wound healing through the regulation of keratinocytes migration and its corresponding signal pathways.

Matrix metalloproteinases (MMPs) are a family of zinc-dependent endo- peptidases capable of degrading different components of the extracellular matrix (ECM) and are essential for the remodeling of pericellular microenvironment required for cell translocation [17]. Although the activation of MMPs results in cancer cell migration and invasion [18], degradation of ECM components by MMPs is also required for keratinocyte migration during wound healing [19]. Human keratinocytes synthesize and secrete mainly MMP-1, MMP-2, MMP-9 and MMP-10 [20]. The gelatinases MMP-9 and MMP-2 contribute to a variety of pathological conditions including cancer, infectious diseases, wound healing, inﬂammation, and vascular diseases [19,21,22]. Increasing evidences suggest that MMP-9 also contributes to keratinocyte migration during wound repair [23,24]. Moreover, plenty of data revealed that metalloproteinases are upregulated by CD9 [25,26]. JNK pathway has been implicated in MMP-9 regulation in human epidermal keratinocytes and HaCaT cells in vitro [27,28] and our previous study revealed that nullification of CD9 upegulates MMP-9 expression in mouse wound healing [14]. However, since CD9 is not only expressed in keratinocytes, but also in other types of cells in skin, it is also unclear whether the observed alteration in JNK or MMP-9 regulation in migrating epidermis in CD9 knockout wounds is directly due to the lack of CD9 in keratinocytes or indirectly due to the influences by other skin cells lacking CD9.

In the present study, we hypothesized that the downregulation of CD9 promotes keratinocyte migration and proposed the mechanism by which the downregulation of CD9 promotes keratinocyte migration through JNK and MMP-9 pathway. Our results revealed that tetraspanin CD9 was downregulated in migrating keratinocytes at wound margin *in vivo* and *in vitro*. CD9 silencing promoted keratinocyte migration, accompanied with the elevation of MMP-9 activity and expression in keratinocytes through JNK pathway. Furthermore, the migration of CD9-silenced keratinocytes was significantly inhibited by MMP-9 activity inhibitor. Thus, our findings suggest that downregulation of CD9 is beneficial for keratinocyte migration *in vitro* and the upregulation of MMP-9 through JNK pathway is involved in the process.

## Results

### Tetraspanin CD9 was downregulated in keratinocytes at wound margin *in vivo* and *in vitro*

**Figure 1:**
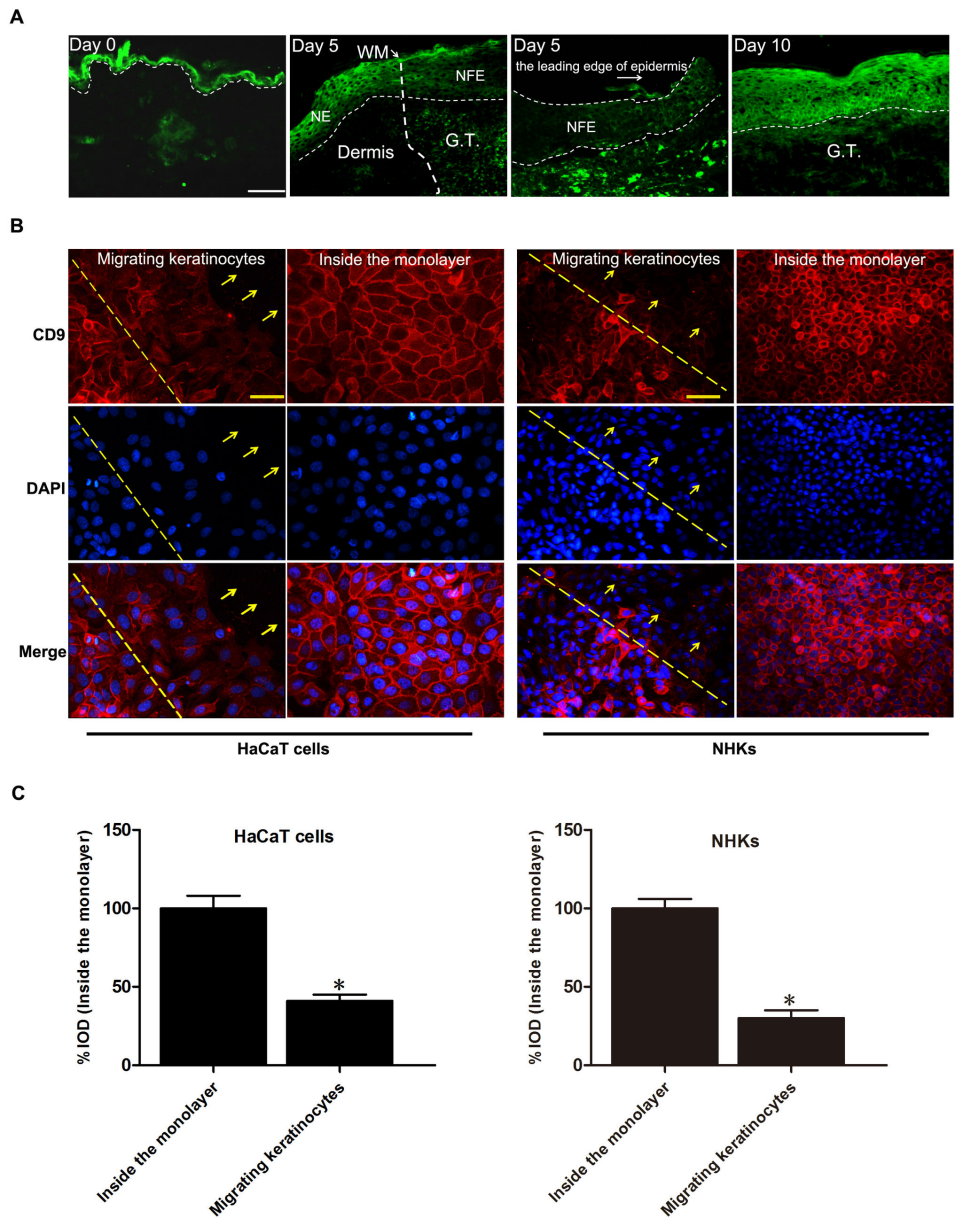
Downregulation of CD9 in keratinocytes at wound margin *in* *vivo* and *in* *vitro*. (A) Immunofluorescence staining of CD9 in normal skin (Day 0) and wounded skin (Day 5 and Day 10) sections obtained from wild-type mice showing downregulation of CD9 in migrating epidermis during wound re-epithelialization. Wounds were fully re-epithelialized on day 10. Scal bar: 100μm. GT, granulationtissue; WM, wound margin; NE, normal epidermis; NFE, newly formed epidermis. Narrow-dotted line: interface between epidermis and dermis or leading edge of migrating epidermis (Day 5, right panel). Wide-dotted line: wound margin. (B) Immunofluorescence analysis of CD9 in HaCaT cells and normal human keratinocytes (NHKs) wounded using a micropipette tip (red labelling, 18 hours after wounding). Nuclei were stained with DAPI (blue). In the upper panel, the dotted line corresponds to the wound incision and the arrows indicate the direction of migrating keratinocytes. The lower panel shows images depicting the expression of CD9 in keratinocytes far from the wounded area of the cell monolayer. Scalebar: 50 μm. (C) Graphs to the right represent the corresponding % Integral optical density (IOD) of CD9 (red) (values were normalized as percentage after comparison with the keratinocytes inside the monolayer, which were set to 100%; n = 3). Results showed that CD9 expression in migrating keratinocytes was significantly downregulated. *P<0.05 vs. Inside the monolayer.

Mice were punched as described in the methods section to detect the change of CD9 expression during wound repair, and skin histological sections were obtained after punch injury. CD9 was highly expressed in the normal epidermis of skin (Day 0, Figure 1A), but markedly downregulated in the newly formed migrating epiderms post-wounding (Day 5, Figure 1A). When the wound developed to re-epithelialization, the expression of CD9 in epidermis of skin was elevated to normal level (Day 10, Figure 1A). We also observed that CD9 was downregulated in migrating keratinocytes at the wound edge in a scratch wound of confluent HaCaT cells and normal human keratinocyte (NHK) monolayers *in vitro* compared to that of the unscrathed part of the monalayer that was away from the scratch site (Figure 1B and 1C).

### Downregulation of CD9 promoted keratinocytes migration

**Figure 2:**
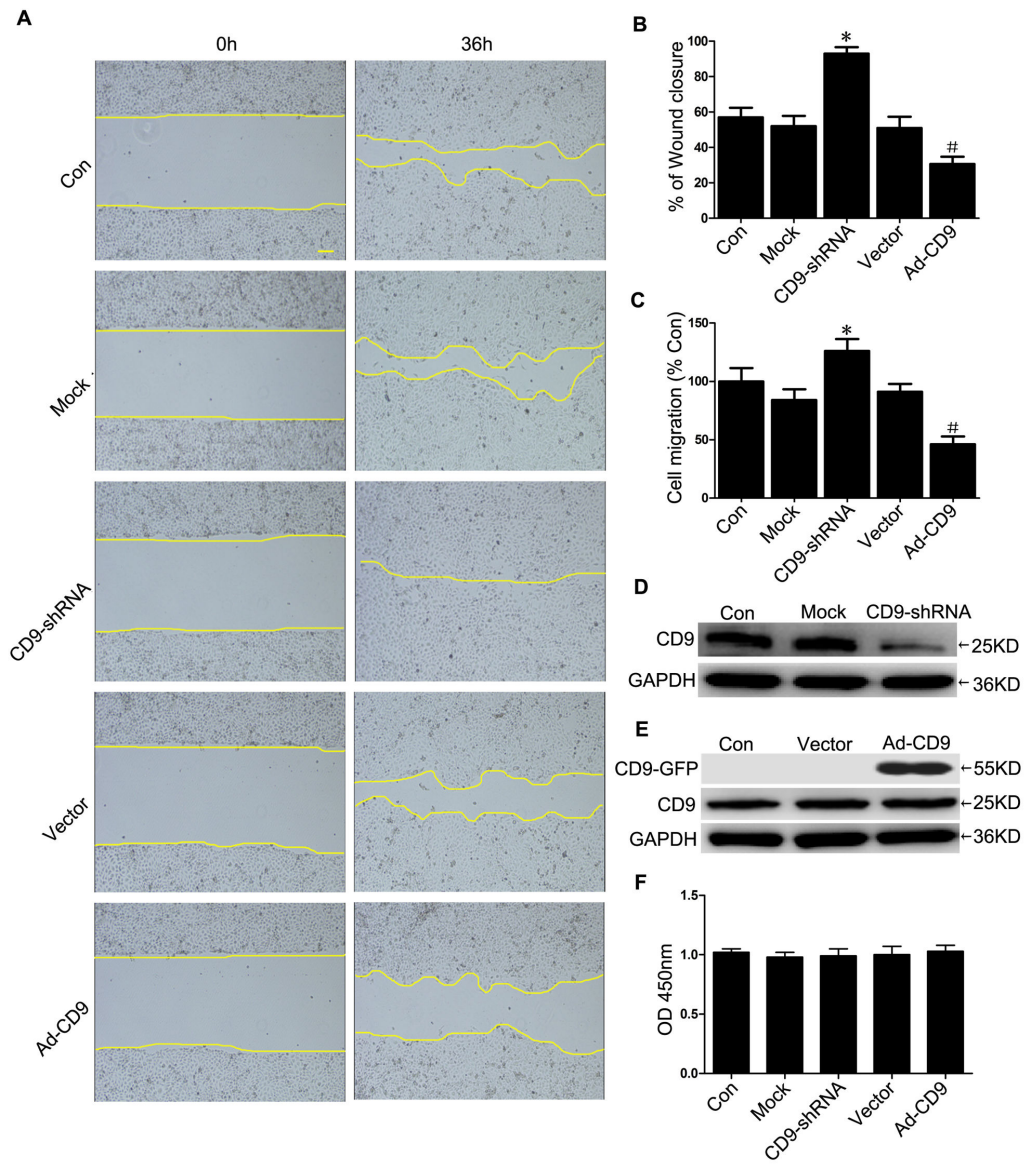
Downregulation of CD9 promoted HaCaT cells migration. (A) HaCaT cells uninfected control (Con) and infected with recombinant adenovirus vectors for silencing of CD9 expression (CD9-shRNA) and overexpressing CD9 (Ad-CD9) and negative vectors (Mock and Vector group) were scratch wounded with a micropipette tip (200 µL) and recorded by phase-contrast microscopy connected to a digital camera at time 0 and 36 hours (n = 3). Scalebar: 200 μm. (B) The wound closure was illustrated by showing the the area covered by keratinocytes immediately and 36 hours after wounding. The panel represents the quantification of the CD9 regulation effect on the wound closure as calculated by measuring of the diminution of the wound bed surface upon time using Image J software. (C) Recombinant adenovirus-infected HaCaT cells were seeded in the upper chambers of Transwell inserts. After 24 hours, cells on the lower side of the filter were stained with 0.5% crystal violet and scored in five independent fields (n = 3). (D) and (E) Western blots showed the expression of CD9 in HaCaT cells (n = 5). (F) HaCaT cells proliferation was determined by Cell Counting Kit-8 (n = 3). *P< 0.05 vs. Mock group. #P< 0.05 vs. Vector group.

Recombinant adenovirus vectors for silencing CD9 (CD9-shRNA) and overexpressing CD9 (Ad-CD9) were constructed and used to infect keratinocytes to investigate the impact of CD9 on HaCaT cells migration. After infection for 48 hours, more than 90% of the HaCaT cells were determined to be infected by observing GFP expression using a ﬂuorescent microscope, and the CD9-GFP fusion protein was mainly located in the plasma membrane of HaCaT cells infected by Ad-CD9 (Figure S1). The effective silencing of endogenous CD9 or overexpression of CD9-GFP fusion proteins was conﬁrmed by western blotting and fluorescence-activated cell sorting (FACS) analysis (Figure 2D, 2E and Figure S2).

Then we evaluated the effects of downregulation or overexpression of CD9 on HaCaT keratinocyte migration in a scratch wound assay. Our results showed increased migration activity in CD9-silenced keratinocytes, but decreased migration activity in CD9-overexpressed keratinocytes into the scratched area. After 36 hours, CD9-silenced keratinocytes migrated into over 90% of the scratched area (CD9-shRNA group), while CD9- overexpressed keratinocytes only 30% (Ad-CD9 group), compared with less than 60% by the scramble-infected kerationcytes (Mock group and Vector group) (Figure 2A and 2B). No difference in cell proliferation was detected in these groups (Figure 2F). Thus, cell proliferation can be excluded as a possible explanation for migratory capacity alternation of CD9-regulated keratinocytes. The regulatory role of CD9 in HaCaT cells migration was further confirmed by the transwell migration assay, in which the number of cells migrating through the porous membranes of the transwell chambers were increased by 50% in CD9-silenced HaCaT cells, but decreased by 49.3% in CD9-overexpressed HaCaT cells, compared with the mock vector group (Figure 2C). Similar role of CD9 in migration was also observed in CD9-silenced NHKs, in which cells migration was increased by 26% in the scratch wound assay (Figure S3).

### CD9 inversely regulated MMP-9 activity and expression in keratinocytes

**Figure 3:**
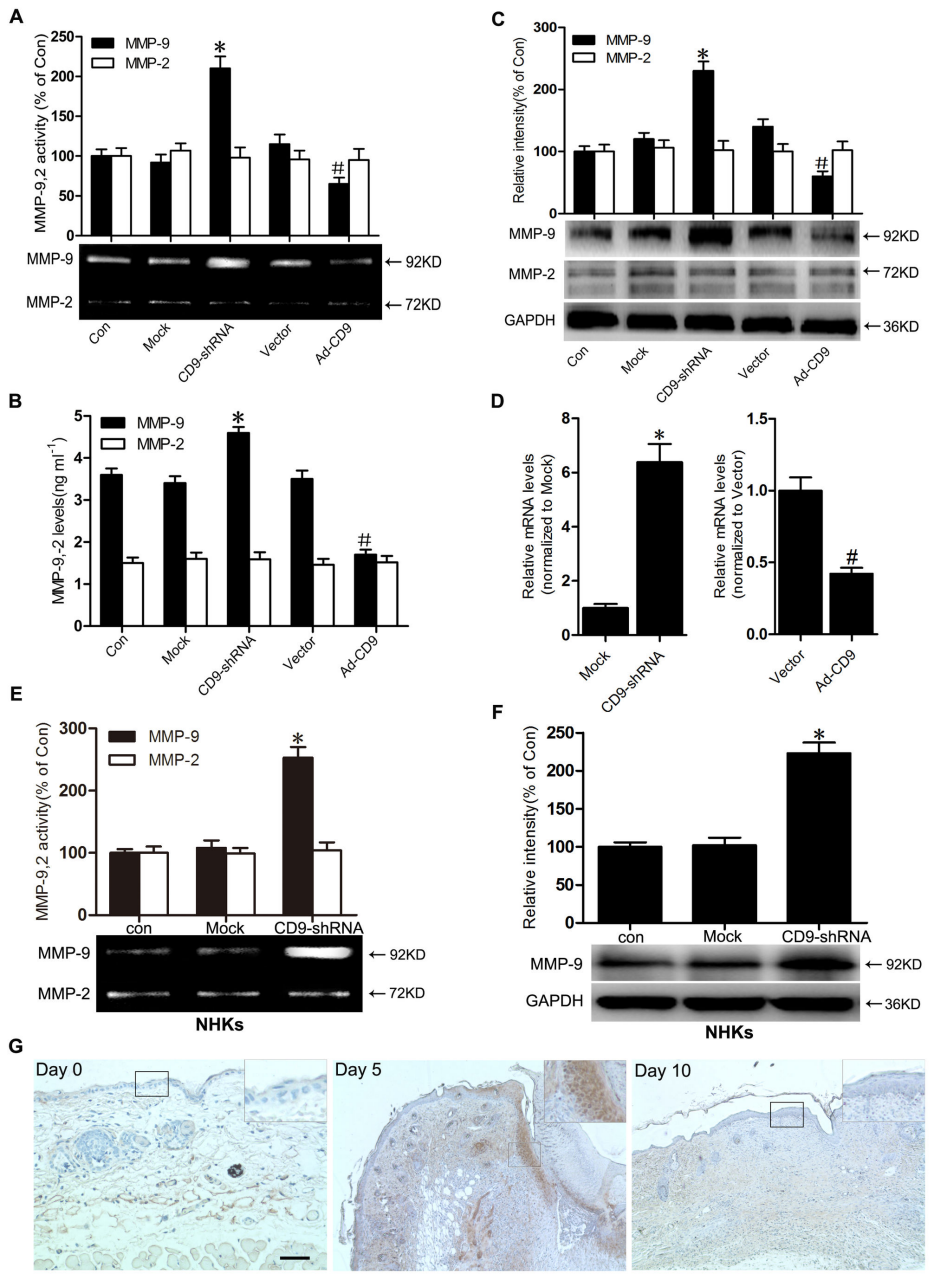
CD9 inversely regulated MMP-9 activity and expression in keratinocytes. (A) HaCaT keratinocytes were infected with recombinant adenovirus vectors for silencing of CD9 expression (CD9-shRNA) and overexpressing CD9 (Ad-CD9) and negative vectors (Mock and Vector group). Results of gelatin zymograms showing the activity of MMP-9 and MMP-2 in the supernatant of cultured HaCaT cells (n = 3). (B) Levels of MMP-9 and MMP-2 were determined in the culture supernatants of cultured HaCaT cells (n = 3). (C) Western blots and the relative integrated signal showed MMP-9 and MMP-2 in cultured HaCaT cells (n = 5). (D) Real-time PCR analysis of MMP-9 mRNA levels from HaCaT cells infected with recombinant adenovirus vectors. The MMP-9 mRNA levels were normalized to Mock group or Vector group. (E) and (F) NHKs infected with recombinant adenovirus vectors for silencing of CD9 expression (CD9-shRNA), and negative vectors (Mock group). Gelatin zymograms (E) showed the activity of MMP-9 and MMP-2 in the supernatant of cultured NHKs (n = 3) and Western blots (F) showed the expression of MMP-9 and MMP-2 in NHKs (n = 5). (G) Immunostaining results showing a stronger signal of MMP-9 in migrating epidermis on Day 5 post wounding than normal skin and on Day 5 post wounding in mice.*P< 0.05 vs. Mock group. #P< 0.05 vs. Vector group.

We assessed the activities of MMP-9 and MMP-2 in supernatants of HaCaT cells and NHKs cultures by zymography. Our results demonstrated that MMP-9 activity was signiﬁcantly higher in CD9-silenced HaCaT cells, but lower in CD9-overexpressed HaCaT cells compared with the mock control cells or mock vector-infected cells (Figure 3A). Similar effects of CD9-silencing on MMP-9 activity was also observed in NHKs (Figure 3E). Using ELISA, we examined the levels of MMP-9 and MMP-2 in the culture supernatants. The data showed that MMP-9 level in the culture supernatants increased in CD9-silenced HaCaT cells, but decreased in CD9- overexpressed HaCaT cells (Figure 3B). However, no signiﬁcant differences in MMP-2 activity and level were detected between CD9-silenced cells and CD9-overexpressed cells. Protein expression of MMP-9 and MMP-2 were analyzed by western blot. The expression of MMP-9 was elevated in CD9-silenced HaCaT cells or NHKs, but attenuated in CD9-overexpressed HaCaT cells. No difference in MMP-2 protein levels was detected between the different experimental cell groups (Figure 3C and 3F). Real-time PCR analysis revealed that the mRNA level of MMP-9 was significantly increased in CD9-silencing keratinocytes, but significantly decreased by CD9- overexpression compared with the scramble-infected control cells (Figure 3D). In addition, We also detected the MMP-9 expression in migrating HaCaT keratinocytes during wound repair *in vitro*. The result showed that, in opposite to the decrease of CD9 in migrating keratinocytes (Figure 1B), MMP-9 expression was upregulated in migrating keratinocytes at the wound margin after scratching (Figure S4).

To ascertain if the observed downregulation of CD9 in migrating epidermis post-wounding *in vivo* is indeed accompanied by an increase of MMP-9, immunohistochemical staining for MMP-9 before or after wounding was performed. As shown in Figure 3G, MMP-9 was not expressed in normal skin epidermis (Day 0), but was significantly induced after wounding (Day 5). When wounds were close to re-epithelialization by Day 10, the expression of MMP-9 in newly formed epidermis was resilenced to a level comparable with that observed in normal skin. Together with the finding in Figure 1A, this finding indicates a negative corelation between the expression of CD9 and MMP-9 in epidermis during wound healing, which matches our results from the *in vitro* experiments.

### MMP-9 was involved in CD9-regulated keratinocyte migration

**Figure 4:**
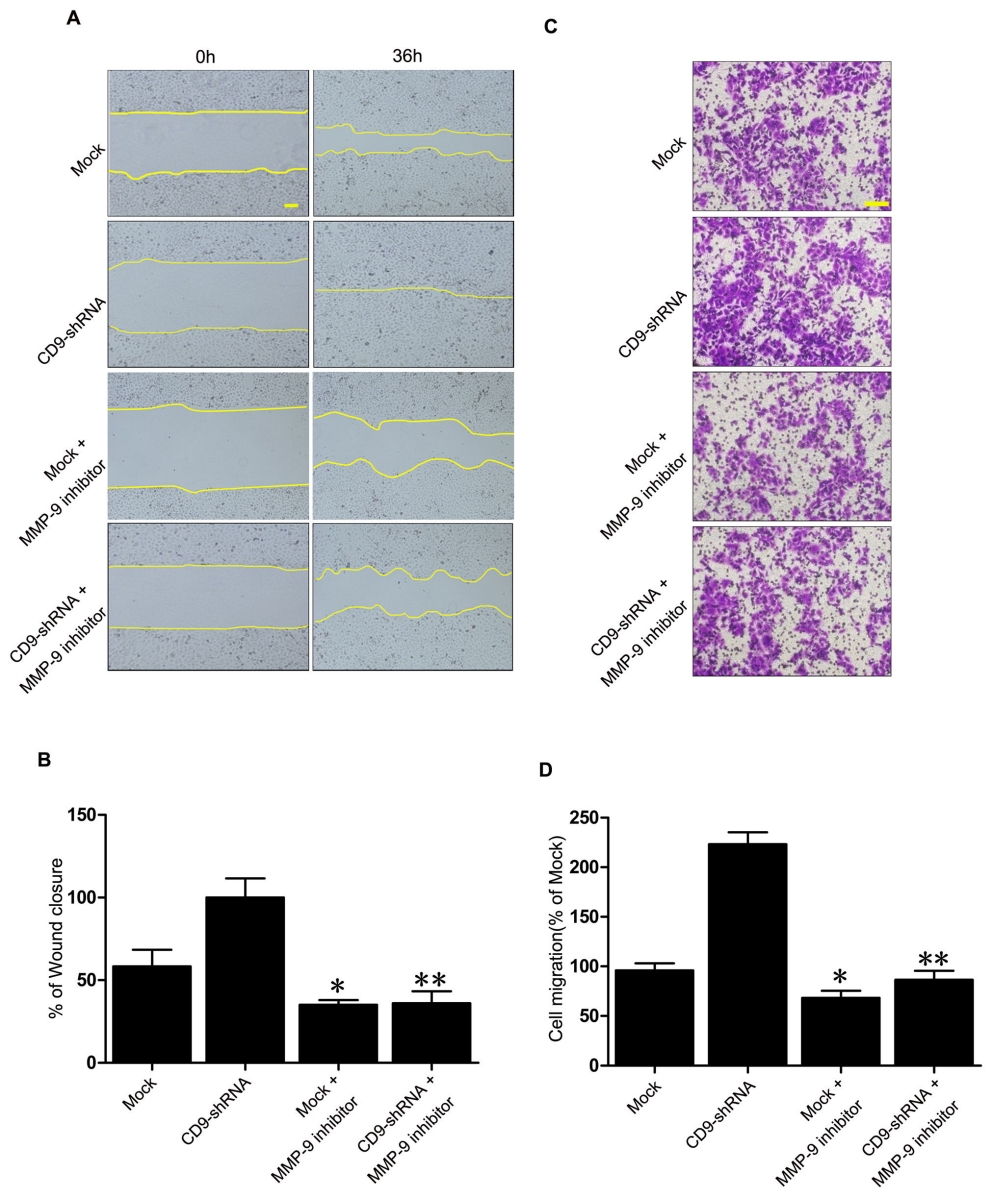
Involvment of MMP-9 in CD9-regulated keratinocyte migration. (A) In the scratch wound assay, Mock-infected and CD9-shRNA-infected HaCaT cells were treated with MMP-9 inhibitor. The area covered by migrating keratinocytes was recorded by phase-contrast microscopy connected to a digital camera at time 0 and 36 hours (n = 3). (B) The wound closure area was calculated by measuring of the diminution of the wound bed surface upon time using Image J software. (C) and (D) HaCaT cells were seeded in the upper chambers of transwell inserts. MMP-9 inhibitor was added to cells at 4°C for 30 minutes prior to plating into wells to assess the effect of activating MMP-9 on cell migration. After 24 hours, cells on the lower side of the filter were stained with 0.5% crystal violet and scored in five independent fields (n = 3). *P< 0.05 vs. Mock group.**P< 0.05 vs. CD9-shRNA group.

Our results demonstrated that CD9 could regulate MMP-9 activity and expression in keratinocytes. We next determined if MMP-9 is involved in the CD9-regulated keratinocyte migration. As shown in Figure 4A and 4B, selective MMP-9 inhibitor significantly impaired the migration of HaCaT cells in a scratch wound. After addition of MMP-9 inhibitor, cell migration was significantly impaired. Wound closure was reduced 2.8-fold in CD9-scilenced keratinocytes, but 1.6-fold in mock-transfected keratinocytes. Moreover, cell migration assay also showed that MMP-9 inhibitor significantly suppressed the migration of CD9-silenced keratinocytes (2.6-fold reduction) and the mock-transfeced keratinocytes (1.4-fold reduction) (Figure 4C and 4D). Hence, our findings suggest that MMP-9 participates in CD9-regulated keratinocyte migration.

### JNK signaling was involved in CD9-regulated MMP-9 production

**Figure 5:**
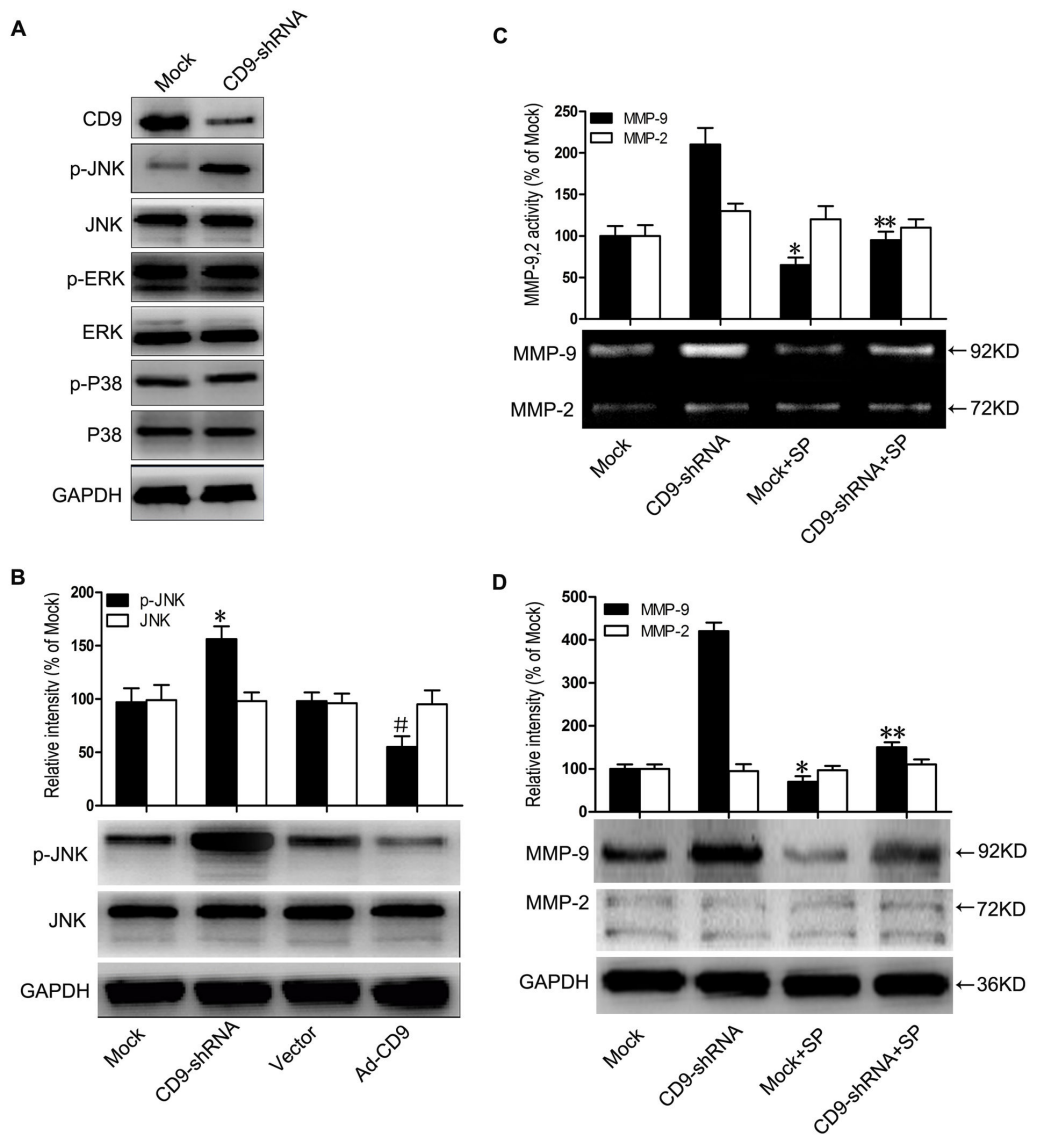
JNK signaling was involved in CD9-regulated MMP-9 production. (A) Representative western blot results showing increased JNK phosphorylation in CD9-shRNA adenovirus-infected HaCaT cells, with no alteration to p38 pathway and ERK pathway. (B) Representative western blot results and data summary of p-JNK and JNK in HaCaT cells infected with CD9-shRNA and Ad-CD9 adenovirus (n = 5). (C) Gelatin zymograms were shown for activity of MMP-9 and MMP-2 in the culture supernatants (n = 3). Mock-infected and CD9-silencing HaCaT cells were treated with JNK signaling inhibitor, SP600125 (SP). (D) Western blots were shown for MMP-9 and MMP-2 (n = 3). *P< 0.05 vs. Mock group. #P< 0.05 vs. Vector group. **P< 0.05 vs. CD9-shRNA group.

To further elucidate the signaling events involved in CD9-regulated MMP-9 expression, we investigated the activation of MAPK pathways in CD9-silenced HaCaT cells, and found a significant increase in JNK phosphorylation (Figure 5A). Phosphorylated JNK (p-JNK) and JNK were tested by immunoblotting in both CD9-silenced and CD9-overexpressed cells. As shown in Figure 5B, the phosphorylation level of JNK was signiﬁcantly decreased in CD9-overexpressed cells but increased in CD9-silenced cells, with levels of total JNK were comparable between the two experiment sets.

 To test whether JNK signaling plays an important role in CD9-regulated MMP-9 expression, HaCaT cells infected with CD9-shRNA vector and Mock vector were both treated with JNK inhibitor SP600125 (10μM). SP600125 at the concentration of 10μM in our experiments inhibited JNK signaling specifically and significantly, but without effects on ERK and p38-MAPK signaling (Figure S5). Our data demonstrated that SP600125 significantly abolished MMP-9 activity in the culture supernatants of CD9-silenced HaCaT cells (Figure 5C). The protein level of MMP-9 in CD9-silenced HaCaT cells was also reduced by JNK inhibition, whereas no change was detected in the protein level of MMP-2 (Figure 5D). Taken together, the results indicate that JNK signaling plays a crucial role in the process of CD9-regulated MMP-9 expression.

## Discussion

In the current study, we revealed that CD9 was markedly downregulated in migrating epidermis during *in vivo* wound repair (Figure 1A). Meanwhile, immunofluorescence results also revealed decreased CD9 staining in migrating keratinocytes (HaCaT cells and NHKs) in a scatch wound model (Figure 1B and 1C). The downregulation of CD9 in migrating keratinocytes *in vivo* and *in vitro* may suggest that a lower level of CD9, compared with unmigrated keratinocytes, is required for the keratinocyte migration. In the *in vitro* experiments, we observed that CD9 silencing promotes wound repair and cell migration of HaCaT keratinocytes on fibronectin, whereas CD9 overexpression has opposite effects (Figure 2). Keratinocytes cover the wound bed by cell migration and proliferation. However, our results did not reveal any significant differences in cell proliferation between CD9-silenced cells and CD9-overexpressed cells (Figure 2F). Thus, we conclude that the effect of CD9 on wound repair in HaCaT cells is predominantly caused by its role in keratinocyte migration. 

 It should be noted that our current finding of the accelerated migration of HaCaT keratinocytes by CD9 downregulation is not consistent with our previous observations in an *in vivo* CD9 knockout mice model, where wound healing was delayed due to the impaired migration of epidermis[14]. Thus, it is possible that primary and immortalized keratinocytes may behave differently with respect to CD9-regulated migration. However, as shown in Figure S3, the downregulation of CD9 also promoted the migration of primary human keratinocytes *in vitro*, thus excluding the possibility that the CD9-regulated migration is different between primary and immortalized keratinocytes. These findings further confirmed that a lower level of CD9 expression is beneficial for *in vitro* keratinocytes migration.

Keratinocyte migration is a coordinated process that involves alteration of cell phenotype and MMPs expression in order to facilitate interaction with provisional matrix [29,30]. MMP-9 and MMP-2 are subclasses of the MMPs due to their gelatinolytic activity and have been shown to participate in wound healing. MMP-9 was reported to play an important role in wound re-epithelialization and keratinocyte migration [23]. Moreover, increasing data revealed that metalloproteinases were regulated by tetraspanins [31,32]. Our results demostrated that the level and activity of MMP-9 secreated by keratinocytes and the expression of MMP-9 in keratinocytes were inversely correlated to the level of CD9, whereas MMP-2 activity and expression did not (Figure 3). Moreover, we confirmed that MMP-9 was involved in CD9-regulated keratinocyte migration by treating keratinocytes with MMP-9 inhibitor (Figure 4). Our ﬁndings concur with previous reports where impaired migration was observed in MMP-9 null cells [24] and MMP-9 shRNA-treated human keratinocytes [33]. 

The healing of an adult skin wound *in vivo* is a complex and orchestrated process which requires the collaborative efforts of many different tissues and cell lineages [15]. Our previous *in vivo* study demonstrated that wound repair is delayed in CD9-knockout mice due to excessive production of MMP-9 [14]. The exact role of MMP-9 in *in vivo* wound healing still remains uncertain. While MMP-9 induction is required for normal wound healing [24], excessive levels of MMP-9 has been shown to disrupt the balance between ECM degradation and formation, and delay the healing process caused by impaired epidermal migration [23,34,35]. MMP-9 is also known to be implicated in the activation of pro-inﬂammatory cytokine [36,37], with increased inflammation possibly delaying wound healing *in vivo* [38]. In addition, it was reported that the inhibition of CD9 impaires vascular endothelial cell migration and angiogenesis during wound repair [39], which may also contritute to our observed healing delay in CD9-null wounds previously. While *in vitro* wound healing shares some common aspects with *in vivo* wound healing, wound healing *in vitro* is quite different in the healing process. In cultured keratinocytes, MMP-9 is expressed despite the absence of the scratched wounds. The increased MMP-9 accelerated or promoted *in vitro* wound healing in this study, which is in accordance with the findings of previous studies [40,41]. The study by Lu et al. revealed that MMP-9 cleaves fibronectin, which contributes to cell migration on fibronectin [42]. MMP-9 is also required for tubular network formation [43] and the actin cytoskeletal organization [44], which are essential to cell migration. Thus, dysregulation of MMP-9 *in vivo*, either over- or under-expression, impairs keratinocyte migration, whereas increased MMP-9 *in vitro* usually promotes keratinocyte migration. These mechanistic discrepancies between the *in vivo* and *in vitro* regulation of wound healing may provide a reasonable explanation for the difference between our previous and current observations: downregulation of CD9 plays an positive role in keratinocyte migration on fibronectin *in vitro* while the healing is delayed in CD9-knockout mice *in vivo.*

Our *in vitro* study showed that JNK signalling was significantly activated in CD9-silenced HaCaT keratinocytes while inactivated in CD9-overexpressed keratinocytes. Moreover, the activity of MMP-9 in culture supernatants and the expression of MMP-9 in CD9-silencing keratinocytes significantly decreased when the JNK activity was inhibited (Figure 5). These findings suggest that JNK pathway is critical for CD9-regulated MMP-9 production. However, the most peculiar feature of tetraspanins is their ability to associate with other tetraspanins, integrins and signaling receptors, thereby forming tetraspanin-enriched microdomains on the cell surface [45]. The best characterized interactions of tetraspanins are those with integrins, which are involved in signalling pathway [46]. It has been reported that CD9 forms a complex with integrin α6, α3, β1, β4 in keratinocytes [47,48], but it is unclear if integrins participate in CD9-regulated JNK pathway activation. Further work is required to elucidate the possible interaction of CD9 with integrins in JNK regulation.

In conclusion, we demonstrated that the downregulation of CD9 is beneficial to keratinocyte migration *in vitro*, and the upregulation of MMP-9 stimulated by JNK pathway is invovled in this process. Our results in this study may potentially introduce a new viewpoint on understanding the underlying mechanism surrounding the biological function of CD9 in keratinocytes.

## Materials and Methods

### Ethics Statement

All animal-based investigations were designed and performed in accordance with the Guide for the Care and Use of Laboratory Animals published by the National Institutes of Health (NIH Pub. No. 85-23, revised 1996). The entire project was reviewed and approved by the Animal Experiment Ethics Committee of the Third Military Medical University in Chongqing, China.

### Cell culture

#### Primary keratinocytes culture

 Isolation of normal human keratinocytes (NHKs) from healthy neonatal foreskin was as described previously [49] with minor modifications. Briefly, the skin was surgically removed and sterilized by sequential washes in PBS (pH 7.4) containing 100 U/mL of pennicillin G and 100 mg/mL of streptomycin (Invitrogen, USA), then cut into 1.0×0.5 cm strips and incubated with 0.25% trypsin/0.04% EDTA solution (Invitrogen, USA) at 4°C overnight. Epidermis was separated by fine forceps. Collected epidermis were then put into a 50 mL Falcon centrifuge tube containing 20 mL of keritinocyte serum free media (Gibco, USA) and gently mixed with a pipette. The cell suspension was filtered, and keratinocytes were collected by centrifugation at 1400 rpm for 5 minutes. 2×10^6^ keratinocytes were seeded into a 100 mm fibronectin (40 μg/mL)-coated plate and cultured with complete K-SFM medium (Gibco, USA) at 37°C for 10 minutes, with unattached cells subsequently were discarded. Keratinocytes were subcultured when reaching 80-90% confluence. Keratinocytes at passage 2 or 3 were used in this study.

#### HaCaT cells culture

HaCaT cells were obtained from Cell Bank of the Chinese Academy of Sciences in Beijing, China. Cells were cultured in RPMI 1640 medium (Hyclone, USA) supplemented with 100 U/ml penicillin, 100 mg/ml streptomycin, and 10% fetal bovine serum (Hyclone, USA). The cells were incubated at 37°C, 5% CO2, and 95% humidity.

### Mice wound-healing experiments and immunohistochemistry

A 3-mm-diameter full-thickness wound was punched on the dorsal midline of 8-week-old BALB/c male mice using a biopsy punch. Wounded areas surrounded by unwounded skin were dissected at day 0, 5 and 10 after injury, fixed in paraformaldehyde and embedded in paraffin. Wound specimen sections were deparaffinised, rehydrated, and antigen retrieval was performed by microwave treatment at 650 W for 6 minutes in citrate buffer pH 6.0. For immunofluorescence staining of CD9, wound specimens were incubated with rabbit anti-CD9 primary antibody (1:100 dilution; Santa Cruz, USA) at 4°C overnight followed by proper wash and further incubation with Alexa Fluor® 488 Goat anti-rabbit (1:100 dilution; Invitrogen, USA) at 37°C for 1 hour. Fluorescence was observed using a Leica confocal microscope (Leica Microsystems, Wetzlar, Germany). For immunoenzyme staining of MMP-9, sections were incubated with MMP-9 (1:1000 dilution; Abcam, UK) at 4°C overnight, followed by 30 minutes incubation with biotinylated secondary antibody (1:500 dilution; GE Healthcare Life Sciences, USA) and streptavidin-HRP (1:500 dilution; Zymed Laboratories, USA). The color was developed with DAB peroxidase substrate (DAKO, Denmark) until an optimal color was observed.

### Immunofluorescence microscopy

Cells cultured on fibronectin-coated glass coverslips were ﬁxed in 4% paraformaldehyde for 20 minutes. The fixed cells were incubated with mouse anti-CD9 primary antibody (1:100 dilution;Millipore, USA) at 4°C overnight, washed with PBS and followed by incubation with goat anti-mouse secondary antibody conjugated to cyanine 3 (Cy3; 1:1000 dilution; Beyotime, Shanghai, China) at 37°C for 1 hour. Nuclei were stained with DAPI (Hyclone, USA). The CD9 expression was observed under Leica Confocal Microscope (Leica Microsystems, Wetzlar, Germany). The fluorescence intensity of individual cells was measured and analyzed with Image-Pro Plus 6.0 (Media Cybernetics, Inc. USA).

### Recombinant adenovirus vector for silencing of CD9 expression

The recombinant adenovirus vector for silencing of CD9 expression (CD9-shRNA-GFP) and the negative control adenovirus vector containing non-speciﬁc shRNA (Mock) were purchased from Shanghai GeneChem, Co. Ltd (Shanghai, China). All vectors contained the gene for GFP, which served as a marker. HaCaT cells were infected with CD9-shRNA or the mock vector at a multiplicity of infection of 10 for 48 hours for further experiments. 

### Recombinant adenovirus vector for CD9 overexpression

Ad-CD9-GFP and CD9 mock vector Ad-GFP were produced by Shanghai GeneChem, Co. Ltd (Shanghai, China). The vectors encoded the GFP sequence, which served as a marker gene. All recombinant adenoviruses were tested for transgene expression in HaCaT cells by western blotting. HaCaT cells were infected with Ad-CD9-GFP or a negative vector at a multiplicity of infection of 10 for 48 hours for further experiments.

### Cell scratch wounding assay

Scratch wounding assay, an *in vitro* incisional wound model, was performed as described previously [50]. Six-well plates (BD Biosciences, USA) were incubated overnight in either 1 mL of RPMI 1640 media containing human fibronectin at 40 μg/ml (PROSPEC, Tany TechnoGene Ltd. ,USA). Cells were grown to conﬂuence in the fibronectin-coated plates in serum conditioned RPMI 1640. Scratch wounds were created in confluent monolayers using a sterile p200 pipette tip. Four perpendicular semiopaque marks were placed across each scratch on the external surface of the well to standardize quantitative analysis. After the suspended cells were washed for three times, the wounded monolayers were cultured in RPMI 1640 medium. After incubation for 36 hours, repopulation of the wounded areas was observed under phase-contrast microscopy (OLYMPUS, Japan). Using the NIH ImageJ image processing program, the size of the denuded area was determined at each time point from digital images.

HaCaT cells were treated with MMP-9 inhibitor (Merck Calbiochem, USA) at final concentration 2.0 μM to determine the dependency of migration on MMP-9.

### Cell migration assays

Cell-migration assays were performed using fibronectin-coated polycarbonate ﬁlters (8 mm pore size, Transwell; BectonDickinson, USA). The membrane’s undersurface was coated with ﬁbronectin (40 μg/mL) in PBS at 37°C for 1 hours and blocked with 0.1% BSA in RPMI 1640 at 37°C medium for 30 minutes. The lower chamber was ﬁlled with 600 μL of RPMI 1640 medium supplemented with 10% FBS, and the cells were plated at a density of 3×10^5^ in 100 mL of migration buffer in the upper chamber of triplicate wells followed the incubation at 37°C for 24 hours. Then the transwell inserts were ﬁxed with 10% formalin, stained with 0.5% crystal violet in 10% ethanol for 10 minutes and washed with PBS for three times. Cells in the upper compartment were removed using a cotton wool swab, and the ﬁlter was mounted onto glass slides. Cells from five random fields were counted under 100× magnification. Mean cell numbers for each sample were from triplicate inserts.

### Cell proliferation assay

The HaCaT cells were seeded at 2 × 10^3^/well in 96-well plates in RPMI 1640 medium supplemented with 10% FBS. Cell proliferation was determined by Cell Counting Kit-8 (CCK-8; Dojindo Molecular Technologies, Rockville, MD, USA) according to manufacturer's instruction. CCK-8 assay is a colorimetric assay for the determination of the number of viable cells by producing a water-soluble formazan dye upon reduction in the presence of an electron carrier using tetrazolium salt WST-8. After inoculating cell suspension in a 96-well plate, the plate was pre-incubated for 36 hours in a humidified incubator (at 37°C, 5% CO2). CCK-8 solution (10 μL) was added to each well of the plate and then the plate was incubated for 1 hour. At last, the absorbance was measured at 450 nm using a microplate reader (Thermo, USA). The experiment was repeated three times.

### Gelatin zymography

Subconfluent cultures of 6×10^5^ cells were cultured in 6-well plates containing serum-conditioned media overnight followed by 24-hours in 1 mL of serum-free media. Secreted MMP-9 and MMP-2 activity were investigated in harvested supernatants. The JNK inhibitor, SP600125 at final concentration 10 μmol/L (Beyotime, Shanghai, China) was added to these cultures and incubated at 37°C for 30 minutes before serum-free media was added. For each gel, samples containing equal amount of proteins determined by RCDC protein assay were mixed with nonreducing sample buffer. Aliquots of 20 μL of each sample were diluted 3:1 with nonreducing buffer. The samples were electrophoresed on 10% polyacrylamide gels copolymerized with 1 mg/mL gelatin. After electrophoresis, the gel was washed twice in 2% Triton-X-100 (Sigma, USA) for 10 minutes and then incubated in incubation buffer at 37°C overnight. After incubation, the gel was stained with 0.5% Coomassie Brilliant Blue (Beyotime, Shanghai, China) and destained with gel-clear destaining solution. Gelatinolytic activities were detected as transparent bands against a background of Coomassie Brilliant Blue-stained gelatin. The signal intensities of the products were measured using SCIONIMAGE analysis software. 

### Determination of MMP-9 and MMP-2 levels

Levels of MMP-9 and MMP-2 in the culture supernatants were determined with enzyme-linked immunosorbent assay (ELISA) kits (USCN, USCN life science, Wuhan, China) according to the manufacturer’s instructions. Cell supernatants were prepared the same as in zymographic assay.

### Western blot analysis

Cells were washed with ice-cold phosphate-buffered saline (PBS), harvested in 70-200 μL of 1× loading buffer on ice, and homogenized. Lysates were sonicated for 4 seconds, and separated by centrifugation at 4°C and 14000×g for 2 minutes. Protein concentration was determined by RCDC protein assay kit (Sigma, USA). The lysates containing 10 or 20 μg of proteins were separated on 10% SDS–PAGE gel, and transferred electrophoretically to polyvinylidene diﬂuoride (PVDF) membranes. All the following antibodies were used at 1:1000 dilution except for that anti-p-JNK and anti-p-JNK at 1:500 dilution, the loading control anti-GAPDH at 1:5000 dilution, and the secondary antibody at 1:4000 dilution. The blots were probed using primary antibodies: anti-CD9 (Millipore, USA), anti-MMP-9 and anti-MMP-2 (Abcam, USA), anti-JNK and anti-p-JNK (Santa Cruz Biotechnology, USA), anti-p38, anti-phospho-p38 at Thr180/Tyr182, anti-ERK1/2, and anti-phosphorylated threonine 202 in ERK1 and phosphorylated tyrosine 204 in ERK2 (Cell Signaling, USA). Horseradish peroxidase-conjugated IgG was used as a secondary antibody and GAPDH was used as loading control. Immunocomplexes were imaged using an enhanced chemilumine scence detection kit (Amersham Pharmacia) on the ChemiDoc imaging system (Bio-Rad, USA). The images were quantified with the Quantity One 4.1 software (Bio-Rad, USA). Each experiment was repeated twice, and each group was evaluated three times.

### Real-time PCR of MMP-9 mRNA

Total RNA was extracted from HaCaT keratinocytes with TRIzol reagent (Invitrogen, USA) according to the manufacturer’s instructions. MMP-9 mRNA was subjected to RT-PCR with a Realtime 7500 PCR apparatus (Applied Biosystems) according to the instruction manual (SYBRII Green Realtime PCR; Toyobo). The results were analyzed with Applied Biosystems 7500 system v1.4.0 software. Each experiment was repeated twice, and each group was repeated ﬁve times. PCR conditions were: denaturing once at 95 °C (5 minutes), then 45 cycles at 95 °C (30 seconds) and 63 °C (1 minutes). The sense and antisense primers for human MMP-9 and GAPDH were as follows: MMP-9 (5’-GCCTGCAACGTGAACATCT-3’; 5’-TCAAAGAC CGAGTCCAGCTT-3’) and GAPDH (5’-GGTGGTCTCCTCTGACTTCAACA- 3’; 5’-GTTGC TGTAGCCAAATTCGTTGT-3’).

### Statistical analysis

Data are expressed as mean ± standard deviation (SD). SPSS 12.0 was used for statistical analysis and signiﬁcance was evaluated by one-way ANOVA. P values < 0.05 were considered statistically signiﬁcant.

## Supporting Information

Figure S1 **HaCaT cells were infected with recombinant adenovirus vectors for silencing of CD9 expression (CD9-shRNA), overexpressing CD9 (Ad-CD9) and negative vectors (mock and vector group).** (A) HaCaT cells were infected with negative vector (mock) or CD9-shRNA for 48 hours and then observed under a ﬂuorescence microscope to determine the infection efﬁciency by visualizing expression of the gene for GFP. (B) HaCaT cells were infected with mock vector (Vector) or Ad-CD9 for 48 h and then observed under a ﬂuorescence microscope to determine the infection efﬁciency by visualizing expression of the gene for GFP. Scale bar = 200μm.
(TIF)

Figure S2 **Flow cytometry of CD9 on HaCaT cells infected with recombinant adenovirus vectors for silencing of CD9 expression (CD9-shRNA), over-expressing CD9 (Ad-CD9) and negative vectors (mock and vector group).** HaCaT cells were incubated with anti-CD9 primary antibody. Cells were stained by secondary antibody conjugated with Alexa Fluor® 488, and the results were analyzed by ﬂow cytometry. Secondary antibody alone was used as a control. The x-axis depicts log ﬂuorescence intensity (in arbitrary units), the y-axis depicts cell number.
(TIF)

Figure S3 **Downregulation of CD9 promoted NHKs migration.** (A) NHKs infected with recombinant adenovirus vectors for silencing of CD9 expression (CD9-shRNA) and negative vectors (Mock group) were scratch wounded with a micropipette tip (200µL) and recorded by phase-contrast microscopy connected to a digital camera at time 0 and 36 hours (n = 3). Scalebar: 200 μm. (B) The wound closure was illustrated by showing the the area covered by keratinocytes immediately and 36 hours after wounding. The panel represents the quantification of the CD9 regulation effect on the wound closure calculated by measuring of the diminution of the wound bed surface upon time using Image J software.
(TIF)

Figure S4 **Upregulation of MMP-9 in migrating keratinocytes during *invitro* wound repair.** Immunofluorescence analysis of MMP-9 in HaCaT cells wounded using amicropipette tip (red labelling, 18 hours after wounding). Nuclei were stained with DAPI dye (blue). In the upper panel, the arrows indicate the direction of migrating keratinocytes. The lower panel shows images depicting the expression of MMP-9 in keratinocytes far from the wounded area of the cell monolayer. Scale bar: 50 μm.
(TIF)

Figure S5 **Effect of SP600125 on the JNK, ERK and P38-MAPK signaling.** Western blot analysis of phospho-JNK (p-JNK), JNK, phospho-ERK (p-ERK), ERK, phosphor-P38 (p-P38) and P38. The results showed that SP600125 (10 μmol/L) inhibits the JNK signaling significantly, but has no effect on the ERK and p38-MAPK signaling.
(TIF)

Methods S1 (DOCX)
