# Supplementary figures and images for: Downregulation of CD9 in Keratinocyte Contributes to Cell Migration via Upregulation of Matrix Metalloproteinase-9
Source: PLoS One. 2013 Oct 16;8(10):e77806. doi: 10.1371/journal.pone.0077806 (PMC3797697; doi:10.1371/journal.pone.0077806)

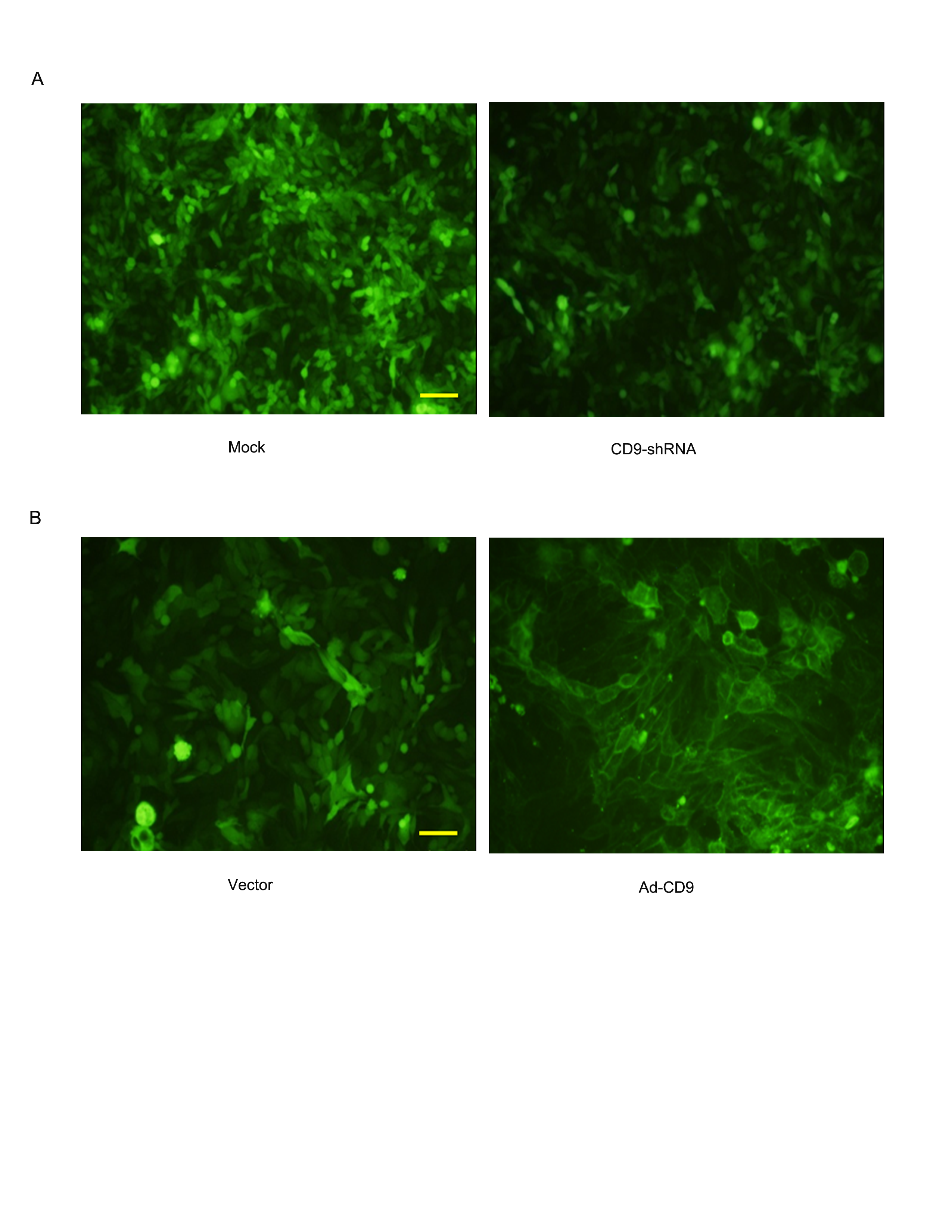

Supplement: Figure S1 — HaCaT cells were infected with recombinant adenovirus vectors for silencing of CD9 expression (CD9-shRNA), overexpressing CD9 (Ad-CD9) and negative vectors (mock and vector group). (A) HaCaT cells were infected with negative vector (mock) or CD9-shRNA for 48 hours and then observed under a ﬂuorescence microscope to determine the infection efﬁciency by visualizing expression of the gene for GFP. (B) HaCaT cells were infected with mock vector (Vector) or Ad-CD9 for 48 h and then observed under a ﬂuorescence microscope to determine the infection efﬁciency by visualizing expression of the gene for GFP. Scale bar = 200μm. (TIF) [file pone.0077806.s001.tif]

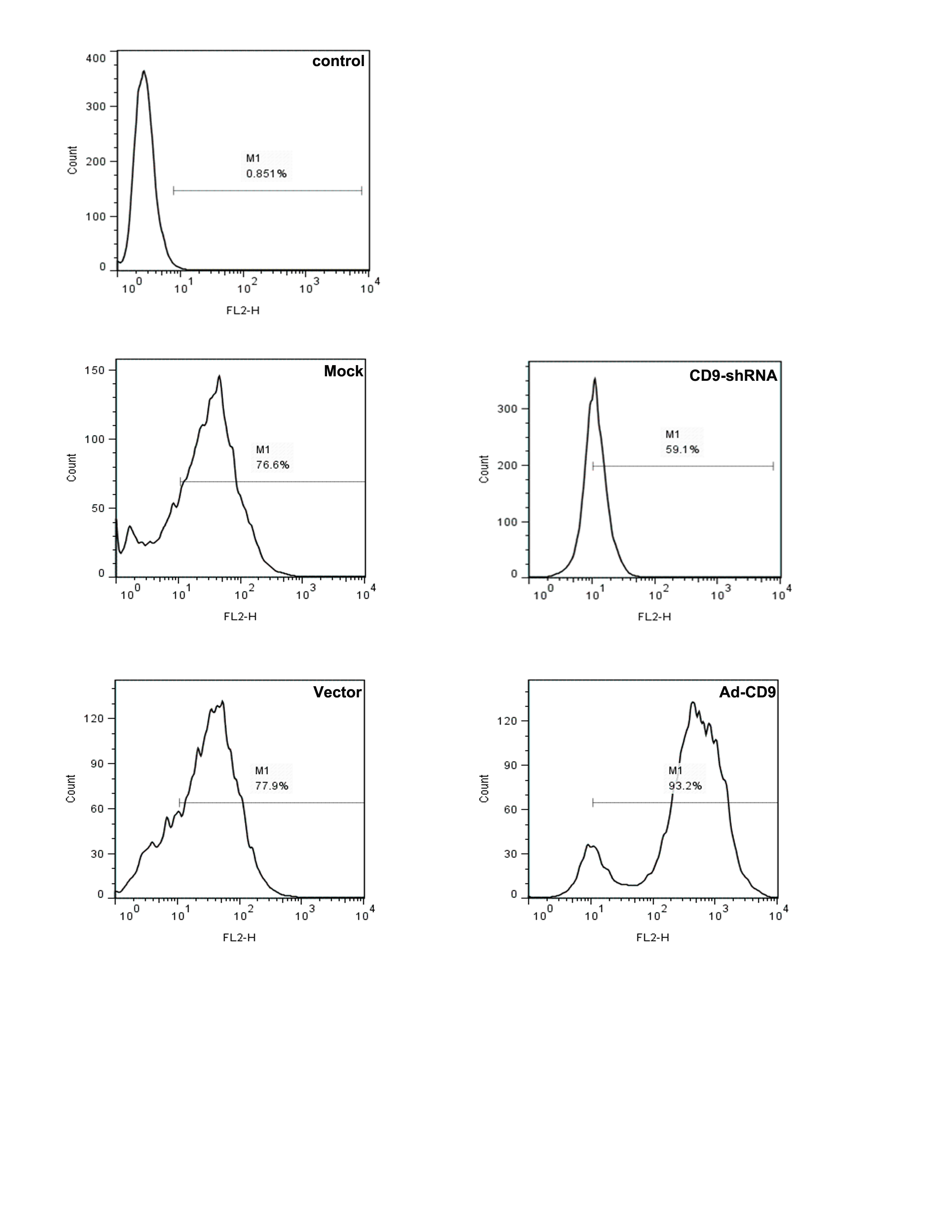

Supplement: Figure S2 — Flow cytometry of CD9 on HaCaT cells infected with recombinant adenovirus vectors for silencing of CD9 expression (CD9-shRNA), over-expressing CD9 (Ad-CD9) and negative vectors (mock and vector group). HaCaT cells were incubated with anti-CD9 primary antibody. Cells were stained by secondary antibody conjugated with Alexa Fluor® 488, and the results were analyzed by ﬂow cytometry. Secondary antibody alone was used as a control. The x-axis depicts log ﬂuorescence intensity (in arbitrary units), the y-axis depicts cell number. (TIF) [file pone.0077806.s002.tif]

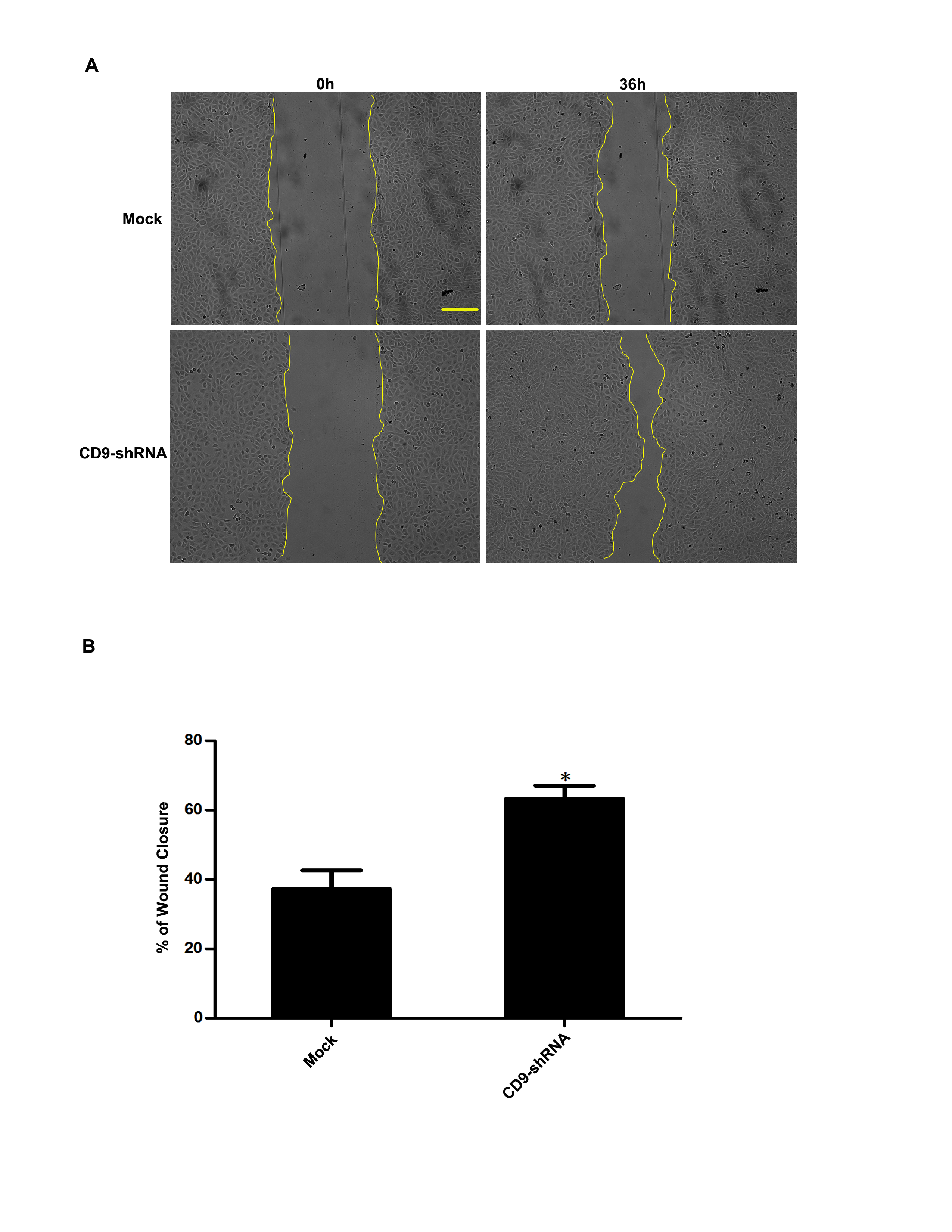

Supplement: Figure S3 — Downregulation of CD9 promoted NHKs migration. (A) NHKs infected with recombinant adenovirus vectors for silencing of CD9 expression (CD9-shRNA) and negative vectors (Mock group) were scratch wounded with a micropipette tip (200µL) and recorded by phase-contrast microscopy connected to a digital camera at time 0 and 36 hours (n = 3). Scalebar: 200 μm. (B) The wound closure was illustrated by showing the the area covered by keratinocytes immediately and 36 hours after wounding. The panel represents the quantification of the CD9 regulation effect on the wound closure calculated by measuring of the diminution of the wound bed surface upon time using Image J software. (TIF) [file pone.0077806.s003.tif]

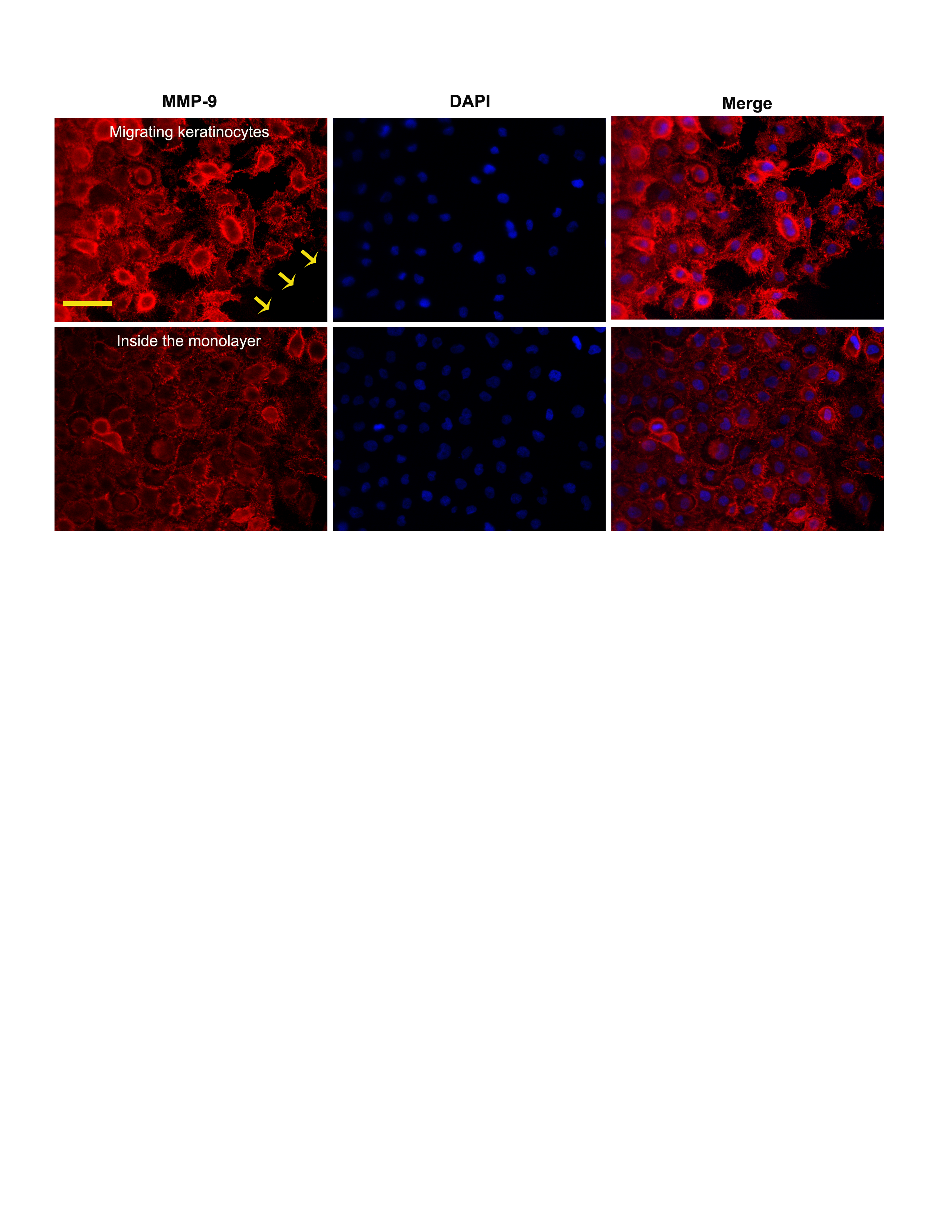

Supplement: Figure S4 — Upregulation of MMP-9 in migrating keratinocytes during invitro wound repair. Immunofluorescence analysis of MMP-9 in HaCaT cells wounded using amicropipette tip (red labelling, 18 hours after wounding). Nuclei were stained with DAPI dye (blue). In the upper panel, the arrows indicate the direction of migrating keratinocytes. The lower panel shows images depicting the expression of MMP-9 in keratinocytes far from the wounded area of the cell monolayer. Scale bar: 50 μm. (TIF) [file pone.0077806.s004.tif]

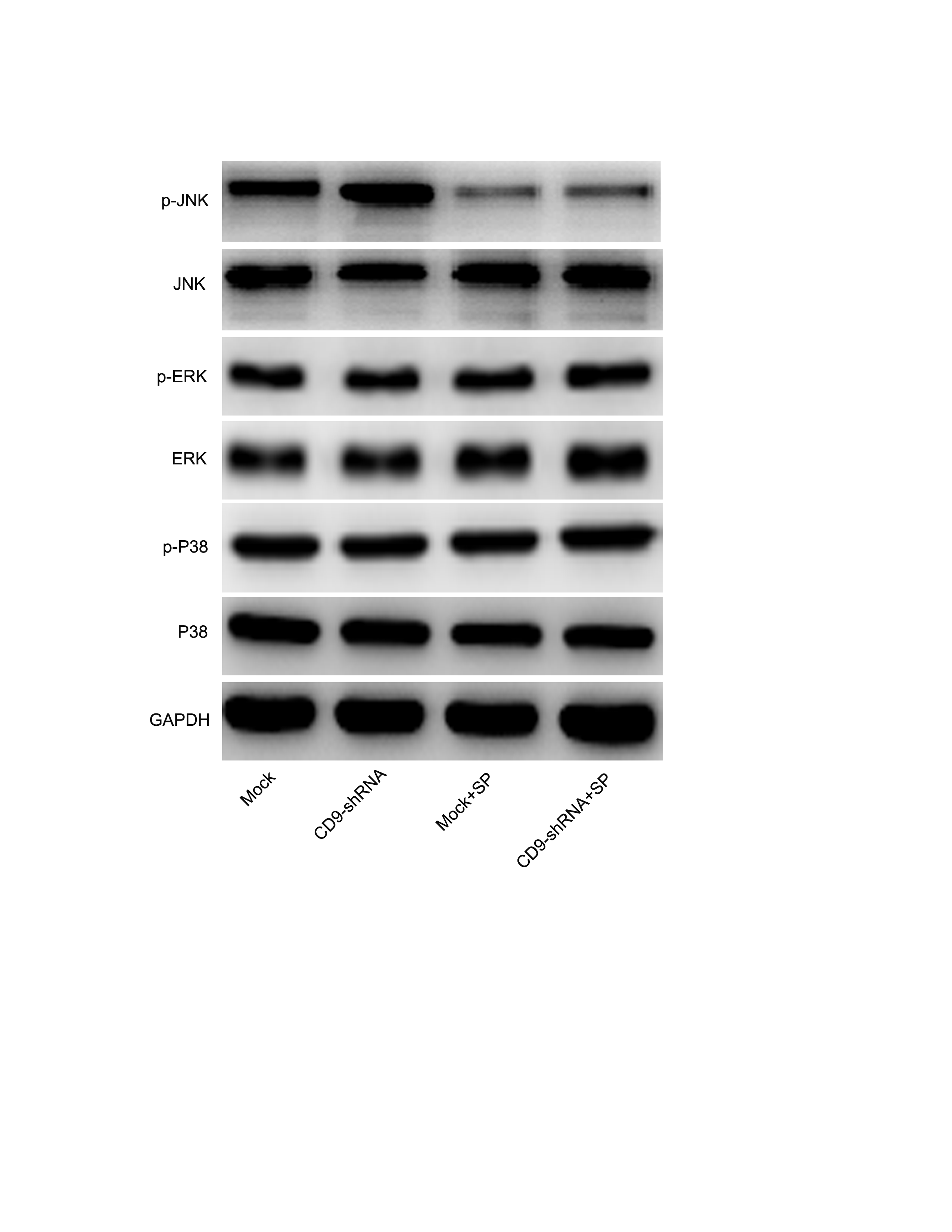

Supplement: Figure S5 — Effect of SP600125 on the JNK, ERK and P38-MAPK signaling. Western blot analysis of phospho-JNK (p-JNK), JNK, phospho-ERK (p-ERK), ERK, phosphor-P38 (p-P38) and P38. The results showed that SP600125 (10 μmol/L) inhibits the JNK signaling significantly, but has no effect on the ERK and p38-MAPK signaling. (TIF) [file pone.0077806.s005.tif]
