# Supplementary material for: Downregulation of CD9 in Keratinocyte Contributes to Cell Migration via Upregulation of Matrix Metalloproteinase-9
Source: PLoS One. 2013 Oct 16;8(10):e77806. doi: 10.1371/journal.pone.0077806 (PMC3797697; doi:10.1371/journal.pone.0077806)
Supplement: Methods S1 — (DOCX) [file pone.0077806.s006.docx]

**Supporting Information**

**Methods S1** Flow cytometry: Subconfluent HaCaT keratinocytes were washed with phosphate buffered saline (PBS), trypsinized and resuspended in FACS buffer (PBS containing 1mM MgCl_2_ and 0.1% BSA). Cells were incubated with rabbit anti-CD9 primary antibody at 4°C for 40 minutes and washed twice with PBS. Alexa Fluor® 488 Goat anti-rabbit secondary antibody (1:100 dilution; Invitrogen, USA) was applied to the cells at 4°C for 30 min. Cells were washed twice with PBS and resuspended in 0.5 mL PBS with 10% fetal bovine serum. Labelled cells were subsequently analysed on a FACScans ﬂow cytometer using Cellquest software (Beckton Dickinson).
